# Access to Family Planning Services Following Natural Disasters and Pandemics: A Review of the English Literature

**DOI:** 10.7759/cureus.26926

**Published:** 2022-07-16

**Authors:** Anusha Adkoli, Savannah Kumar, Itamar D Futterman, Camille A Clare

**Affiliations:** 1 Obstetrics and Gynecology, Rutgers Robert Wood Johnson Medical School, New Brunswick, USA; 2 Obstetrics and Gynecology, New York Medical College, Valhalla, USA; 3 Obstetrics and Gynecology, Maimonides Medical Center, Brooklyn, USA; 4 Obstetrics and Gynecology, Westchester Medical Center/New York Medical College, Valhalla, USA; 5 Obstetrics and Gynecology, State University of New York Downstate Health Sciences University, Brooklyn, USA; 6 Obstetrics and Gynecology, New York Medical College/New York City Health + Hospitals, Metropolitan, Valhalla, USA

**Keywords:** review of literature, earthquake, hurricane, natural disaster, contraception, covid-19, unintended pregnancy, family planning

## Abstract

Background: When natural disasters strike, there is a sudden decrease in access to care due to infrastructure loss and displacement. A pandemic has the similar ability to acutely limit access to care. The relationship between decreased access to care and natural disasters has been previously explored.

Objective: The purpose of this article is to present a focused review of the available and emerging literature regarding the overall impact of natural disasters and pandemics on unintended pregnancy and decreased care in this setting.

Methods: A literature search was conducted on PubMed, Cochrane, Google Scholar, and Embase databases. The search was restricted to studies that were population-based, prospective or retrospective. Only peer-reviewed articles were considered. The search was further restricted to manuscripts in English or officially translated manuscripts. All qualifying papers from which data were extracted were subjected to a quality assessment conducted by two independent investigators (SK and AA). Each investigator reviewed all nine papers relevant to data collection using the Effective Public Health Practice Project (EPHP). MeSH terms were utilized across various databases. Studies were selected that were population-based, prospective or retrospective. Case reports and case series were not used. The primary outcomes were the rates of unintended pregnancy. Secondary outcomes included the use of contraception, short interval pregnancy, and access to reproductive services.

Results: An initial search yielded 74 papers, of which nine papers were reviewed for qualitative data, examining the subjects affected by natural disasters or pandemics. An additional two papers regarding theoretical data and COVID-19 were analyzed. Although there seems to be a rise in unintended pregnancy and more difficulty accessing care following natural disasters and pandemics, there are variations in the rates based on region and event.

Conclusions: The full effects of the COVID-19 pandemic on the rates of unplanned pregnancies will become apparent in the months and years to come. As obstetrician-gynecologists, we must communicate openly with our patients regarding the use of available contraception, sexual education, and family planning services at times of natural disasters and pandemics.

## Introduction and background

Introduction

Between the years 2015 and 2019, it is estimated that each year 121 million pregnancies globally were unintended [1]. This reflects a global unintended pregnancy rate of 64 per 1,000 women of reproductive age [1]. The Centers for Disease Control and Prevention (CDC) notes that unintended pregnancy is one that is “either unwanted, such as that which occurred when no children or no more children were desired, or a pregnancy that is mistimed, such as a pregnancy that occurred earlier than desired.” [2] Attempts to decrease unintended pregnancy focus on increasing access to family planning and contraceptive services as it is believed that these services are vital to providing patients of reproductive age with the necessary contraceptive care, screenings, and tests to make informed health choices regarding pregnancy [3]. The Guttmacher Institute has estimated that in 2016, two million pregnancies were postponed or avoided by the use of contraception prescribed by providers [4]. It is also estimated that these pregnancies could have resulted in approximately one million births and nearly 700,000 abortions [4].

Pandemics and natural disasters have both been shown to decrease the access to family planning services either by the destruction of healthcare facilities and infrastructure or by reduced capacity for care due to social distancing, clinic closures, travel restrictions, or economic slowdowns [5]. Initial findings show that contraceptive supply chains and family planning services have already been disrupted and negatively impacted by COVID-19 [5]. With one in six patients reporting increased difficulty in accessing contraception during the current pandemic [6], it is imperative that providers find a way to meet the demand for family planning services to ensure the health and safety of their patients and prevent unintended pregnancy.

Objectives

When natural disasters occur, there is a sudden decrease in access to care due to infrastructure loss and displacement. A pandemic has a similar ability to limit access to care [5]. The relationship between decreased access to care and natural disasters has been previously explored. The purpose of this article is to present a focused review of the available and emerging literature on the overall impact of natural disasters and pandemics on unintended pregnancy based on patient cohorts pre- and post-exposure. Theoretical data related to the current COVID-19 pandemic and the impact and overall risk of decreased care in this setting are also utilized.

## Review

Methods

Search Strategy

**Figure 1:**
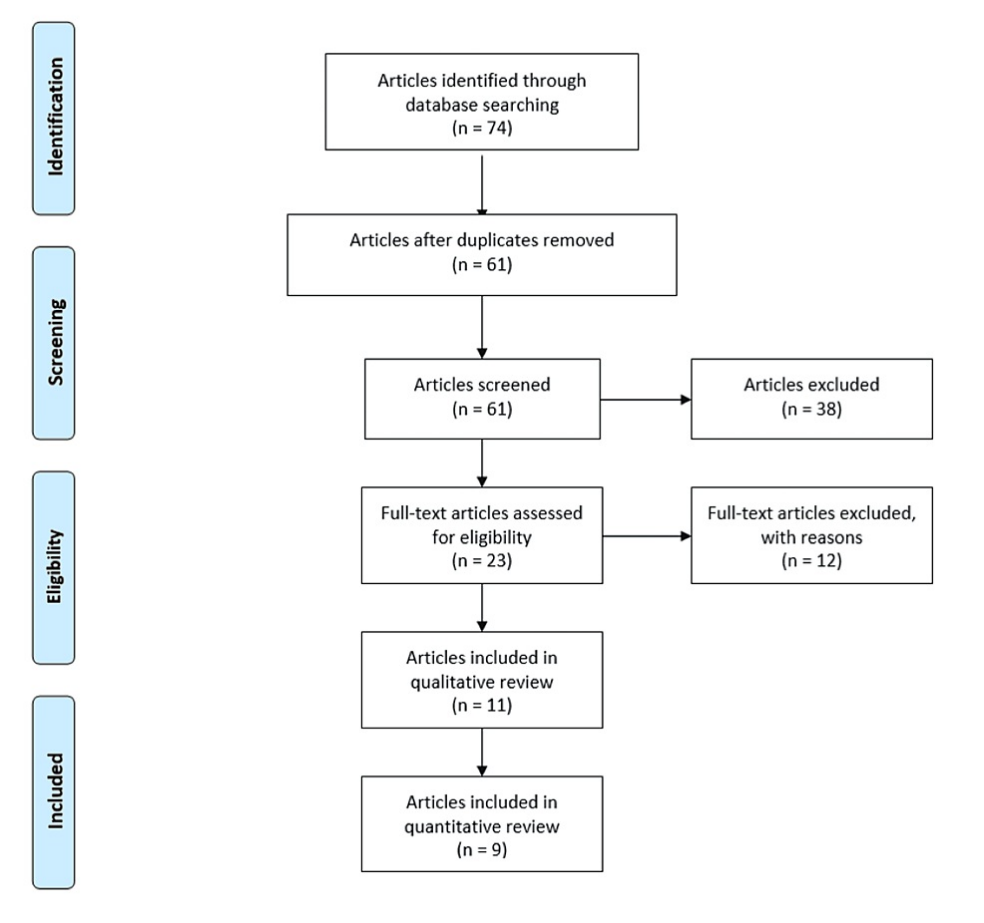
Study selection flow diagram

The following electronic search engines were used to find manuscripts regarding unplanned pregnancy during natural disasters and pandemics: PubMed, Google Scholar, Cochrane, and Embase database. Keyword MeSH terms utilized for the search included “unintended pregnancy,” “unplanned pregnancy,” “natural disasters,” “hurricanes,” “earthquakes,” “floods,” “COVID-19,” “epidemics,” and “pandemics.” This search yielded 74 manuscripts between two investigators (AA and SK). Duplicates were removed, and 61 manuscripts remained. A manual assessment of the references in these 61 manuscripts was performed to further broaden the search and improve accuracy. Additional manuscripts were not identified from this assessment, and the final literature search yielded 61 manuscripts, all of which were screened using eligibility criteria. Of the 61 manuscripts, 11 manuscripts met inclusion criteria and were analyzed. Nine of the 11 manuscripts contained non-theoretical data, while the remaining two manuscripts contained theoretical data regarding the COVID-19 pandemic (Figure 1).

Study Selection and Eligibility Criteria

Manuscripts describing unplanned pregnancy in relation to natural disasters or pandemics were considered eligible for analysis. The search was restricted to studies that were population-based, prospective or retrospective. Only peer-reviewed articles or those published in peer-reviewed journals were considered. The search was further restricted to manuscripts in English or officially translated manuscripts. The exclusion criteria included case reports, case studies, qualitative assessments, and opinion pieces. This review paper was exempt from institutional review board approval.

Study Quality Assessment

All qualifying papers were also subjected to a quality assessment conducted by two independent investigators (SK and AA). Each investigator assessed all nine non-theoretical papers using the Effective Public Health Practice Project (EPHP) [7]. Each paper was assigned a “grade” using a 1-3 system with 1 being “strong” and 3 being “weak” based on factors such as selection bias, study design, confounding variables, dropouts, method of blinding, and withdrawals. A “final quality grade” was assigned to each paper, and the two independent investigators (SK and AA) compared the final quality grade assigned to each paper. Final quality grades were included in Table 1. A third blind reviewer was available for any disagreements between the reviewers; however, there were no discrepancies between the reviewers regarding final quality grades.

**Table 1:** Selected studies EPHP: Effective Public Health Practice Project; aOR: Adjusted odds ratio.

Title	Population	Interventions	Comparison	Outcomes	EPHP Grading
Sexual Activity and Contraceptive Use During Social Distancing and Self-Isolation in the COVID-19 Pandemic	317 women of reproductive age who were listed in the database of the University of Catania, Italy, family planning clinic who were known to be using hormonal contraceptives	COVID-19	Cohort study	50.5% of non-cohabiting or single women had discontinued their long-acting reversible contraceptive (LARC) method while social distancing. However, 46.5% of non-cohabiting or single women had continued their sexual activity, and 14.9% had an unplanned pregnancy [6].	2
The Effect of the Hurricane Katrina Disaster on Sexual Behavior and Access to Reproductive Care for Young Women in New Orleans	Women of reproductive age exposed to Hurricane Katrina	Hurricane	Cohort study	When compared with baseline, after the hurricane, women were less likely to have attended family planning services, to have used birth control, to have >1 sexpartner, to have a vaginal odor or discharge [8].	2
Unwanted Pregnancy After Earthquake in Bam city, Iran	256 randomly pregnant women who were referred to different health centers in Bam city following the earthquake	Earthquake	Cross-sectional study	The prevalence of unwanted pregnancy was high in this study. The assessment showed that a critical step for disaster-affected families is consultation with couples (not only women) to identify the factors relating to unwanted pregnancy [9].	2
Reproductive and Birth Outcomes in Haiti Before and After the 2010 Earthquake	Women who gave birth and were affected by the Haiti earthquake	Earthquake	Cohort study	Post-earthquake births were less likely to be wanted and more likely to be born after a short interpregnancy interval. Earthquake exposure was associated with an increased likelihood of a child being born too small [10].	1
Unintended Pregnancy During COVID-19 Pandemic Among Women Attending Antenatal Care in Northwest Ethiopia: Magnitude and Associated Factors	Women during the COVID-19 pandemic	COVID-19	Cohort study	The magnitude of unintended pregnancy during the COVID-19 pandemic among women attending antenatal care was found to be 47.17% (42.2%-52.2%). Women not exposed to community education (aOR = 2.2; 95% CI: 1.1-4) were significantly associated with unintended pregnancy [11].	2
Effect of the September 2009 Sumatra Earthquake on Reproductive Health Services and Millennium Development Goal 5 (MDG) in the City of Padang, Indonesia	Women aged 15-19 who received services at selected facilities at least twice before and after the earthquake	Earthquake	Cohort study	The previous rate of improvement in maternal and child mortality was slowed down, whereas stillbirths increased after the earthquake. Thus, there is a need to speed up the recovery to achieve the local MDGs [12].	1
Change in Contraceptive Methods Following the Yogyakarta Earthquake and Its Association With the Prevalence of Unplanned Pregnancy	Women of reproductive age exposed to Yogyakarta earthquake	Earthquake	Cohort study	The prevalence of unplanned pregnancy was significantly higher in a group of participants who had difficulty accessing contraceptive methods compared to a group that did not [13].	2
Effect of the COVID‐19 Pandemic on Female Sexual Behavior	Women during the COVID-19 pandemic	COVID-19	Cohort study	Use of contraception during the pandemic significantly decreased among participants compared with before the pandemic (24 vs 10, p = 0.004). Menstrual disorders were more common during the pandemic than before (27.6% vs 12.1%, p = 0.008) [14].	2
Peripartum Outcomes Before and After Hurricane Harvey	Women delivering before and 280 days after Hurricane Harvey	Hurricane	Cohort study	Despite having fewer at-risk baseline characteristics, gravid patients delivering after landfall by Hurricane Harvey had a significantly higher likelihood of adverse outcomes as did their neonates [15].	1

Outcomes Assessed in Literature Review

The primary outcomes were the rates of unintended pregnancy. These values were extracted from papers that collected data via self-report from patients and theoretical data projecting the effects of decreased access to care during the COVID-19 pandemic. Other outcomes included difficulty accessing reproductive health services, the use of birth control overall, new use of intrauterine devices (IUDs), new use of oral contraceptives, the desire to become pregnant, and frequency of sexual intercourse. The adverse effects studied were neonatal morbidity, short interpregnancy interval, vaginal infections/vaginal discharge/odor, and menstrual disorders.

The predictions for the COVID-19 pandemic summarized were derived from recent data from two studies utilizing a predictive mathematical model that estimated outcomes based on either a three- or 12-month family planning service disruptions, a 10% decline in family planning services overall, or a 10% shift in abortion care from safe to unsafe abortions [5,16].

Results

Characteristics of Selected Studies

Nine studies met inclusion criteria and are described in Table 1. Four studies described earthquakes, two described hurricanes, and three described current COVID-19 data. There were eight cohort studies and one cross-sectional study. Two additional papers described theoretical COVID-19 predictions.

Unintended Pregnancy

Unintended pregnancy in those exposed to the COVID-19 pandemic or natural disasters was assessed. Data relating unintended pregnancy to exposure to natural disasters was specifically found in relation to Hurricane Katrina in the United States in 2005, the 2003 earthquake in Bam city, Iran, and the 2010 earthquake in Haiti. Studies have shown the rates of unintended pregnancy within subsets of the population in these countries to be 47% in the United States in 2019 [17], 27% in Iran in 2019 [18], and 61.2% in Haiti in 2018 [19].

In the aftermath of Hurricane Katrina, one study reported that 4% of their 55-person cohort experienced an unintended pregnancy because of loss of care secondary to the Hurricane Katrina displacement (p = 0.25) [8]. Similarly, in Bam city, Iran, a study found that 28.1% of the 256 pregnancies assessed were considered to be unintended in the province prior to the natural disaster compared to a previous baseline of 18.6% of unintended pregnancies [9]. A study of the effects of the 2010 Haitian earthquake found that births occurring post-earthquake were less likely to be wanted compared to births prior to the 2010 disaster [10].

One study regarding COVID-19 found 15 (14.9%) unintended pregnancies in a cohort of 101 non-cohabitation/single subjects in Catania, Italy [6]. The incidence of unintended pregnancy in Italy was noted to be 28 per 1,000 women aged 15-49 between 2015 and 2019 [20]. Incidence within the Catania community specifically is not available. In northwest Ethiopia, a recent study found that 47.17% of their 424-person cohort receiving antenatal care reported their pregnancies to be unintended during the COVID-19 pandemic [11]. This study pointed to specific factors such as exposure to community education and lack of healthcare support as factors that increased the likelihood of unintended pregnancy during the COVID-19 pandemic [11]. The incidence of unintended pregnancy in Ethiopia was noted to be 79 per 1,000 women aged 15-49 between 2015 and 2019 [21].

Healthcare Access and Birth Control

Difficulty accessing reproductive health services, the desire to become pregnant, and the use of birth control in those exposed to natural disasters or the COVID-19 pandemic were studied. Difficulty accessing reproductive health services after a natural disaster was noted in one study based on the 2009 Sumatra earthquake in Indonesia, which found that 552/667 (82.8%) respondents reported an increased difficulty in accessing reproductive health services post-earthquake compared to 11/667 (1.6%), who reported no change in accessibility (OR 0.11; 95% CI: 0.08-0.14) [12]. Studies exploring the use of birth control found that there was an increase in the number of women who were not using birth control in the populations exposed to the 2006 Yogyakarta earthquake in Indonesia and Hurricane Katrina in the United States [8,13], while the use of contraception, in general, showed a significant decrease after exposure to the Yogyakarta earthquake [13].

The use of and changes in contraception use were primarily assessed in those exposed to the Sumatra and Yogyakarta earthquakes. A statistically significant decrease in the use of oral contraceptive pills (OCPs) and the number of new IUDs was shown after exposure to the disasters in Sumatra and Yogyakarta, while no relevant change was observed in the continued use of implants, injections, or non-hormonal birth control methods, such as condoms and coitus interruptus [12,13].

During the COVID-19 pandemic, one observational study in Turkey found that of the 58 subjects surveyed, 24 (41.3%) used contraception prior to the pandemic versus only 10 (17.2%) during the pandemic [14]. This value was found to be a statistically significant decrease in the use of contraception (p = 0.004). A statistically significant decrease in the desire to become pregnant as well as an increase in the frequency of sexual intercourse was also found in this cohort [14].

In a survey study in Italy during the COVID-19 pandemic, all 216 married or cohabitating patients continued their method of birth control, both long-acting and short-acting contraceptives, during the pandemic, and none had an unplanned pregnancy [6]. It was also found that 51/101 (50.5%) single women self-discontinued their short-acting reversible contraceptives; however, 47/101 (46.5%) continued with sexual activity despite social distancing rules leading to the 15/101 (14.9%) unintended pregnancies mentioned above [6].

Neonatal Morbidity: A Secondary Outcome

An increase in neonatal morbidity was demonstrated in a cohort study examining the effects of Hurricane Harvey in the United States in 2017 with a rate of 1965/25337 (7.8%) before and 456/3842 (11.9%) after the hurricane (aOR 1.52 [1.34-1.71]) [15]. This is for the composite neonatal morbidity; not all individual components were found to be statistically significant. The authors defined neonatal morbidity as "including any of the following: 5-minute Apgar score ≤ 3, respiratory distress syndrome, the use of ventilator or continuous positive airway pressure, suspected newborn sepsis, seizures, stillbirth, or neonatal death.” [15] Components found to be significant were respiratory distress syndrome (aOR 1.75 [1.44-2.13]) and suspected sepsis (aOR 1.54 [1.34-1.77]) [15]. Short interval pregnancy during the Haitian earthquake was noted as an increase (p < 0.001) (<12 months) post-earthquake from 690/7280 (9.5%) to 1086/7280 (14.9%) [10].

Abnormal Uterine Bleeding and Vaginal Infections

Menstrual disorders, defined as abnormal uterine bleeding or amenorrhea during the COVID-19 pandemic, were shown to have increased after the start of COVID-19 [6]. A study conducted in Turkey during the COVID-19 pandemic found menstrual disorders to be 7/58 pre-pandemic (12.1%) and 16/58 (27.6%) during the pandemic (p = 0.008) [11]. Furthermore, vaginal disorders were determined in the setting of COVID-19 in Turkey and Hurricane Katrina in the United States through two studies using patient-reported questionnaires. These showed a decrease in vaginal infection/vaginal discharge/odor post-pandemic/hurricane. The COVID-19 study had a report of 11/58 (18.9%) patients pre-pandemic and 8/58 (13.8%) patients during the pandemic (p = 0.143) [11]. The Hurricane Katrina study compared 24/55 (43.6%) patients pre-hurricane to 9/55 (16.4%) post-hurricane (p = 0.001) [8]. Vaginal infections were not defined in these studies.

COVID-19 Predicted Outcomes

Two studies utilized predictive algorithms to describe the trajectories of unplanned pregnancy and decreased contraception use due to the COVID-19 pandemic. Additional predictive outcomes included additional maternal deaths, unsafe abortions, and neonatal deaths. The compiled data between the two studies are described in Table 2. A recent study by the United Nations Population Fund (UNFPA) utilized a graded service disruption model to illustrate various outcomes based on the level of disruption (low, medium, and high) and length of disruption (3, 6, 9, and 12 months). It showed that the highest level of a 12-month high service disruption of family planning services could lead to 15,000,000 unplanned pregnancies worldwide [3]. Similarly, a separate study by Riley et al. utilized 2019 sexual and reproductive data from 132 low-to-middle-income countries, up-to-date contraceptive methods/needs, coverage received, and population data to estimate that a 10% decline in family planning services would lead to 15,401,000 unplanned pregnancies worldwide [16].

**Table 2:** Predictions for COVID-19 pandemic

	Decreased contraception use	Unintended pregnancy	Additional maternal deaths	Additional unsafe abortions	Additional neonatal complications	Additional neonatal deaths
3-month low service disruption	13,000,000 women unable to use [3]	325,000 [3]				
12-month high service disruption	51,000,000 [3]	15,000,000 [3]				
10% decline in services	48,558,000 [16]	15,401,000 [16]	28,000 [16]		2,591,000 [16]	168,000 [16]
10% shift in abortion safe to unsafe			1,000 [16]	3,325,000 [16]		
Average of 12-month disruption and 10% decline	49,779,000 [3,16]	15,200,500 [3,16]				

These separate studies each found similar projections of unplanned pregnancy rates. The United Nations Population Fund (UNFPA) described that the rates of contraception use were predicted to decline by 51 million people with a 12-month high service disruption, and Riley et al.’s study found that the rates were predicted to decline by approximately 48.5 million [3,16]. Riley et al.’s study further predicted that with a 10% decline in services or a 10% shift from safe abortion to unsafe abortion, there would be additional maternal deaths, unsafe abortions, neonatal complications, and neonatal deaths [16]. The two studies with different algorithms and grading models, however, both demonstrated similar predictions for unplanned pregnancies with an average of approximately 49.8 million with decreased contraception use and approximately 15.2 million unintended pregnancies [3,16].

Discussion

Infrastructure and supply chain disruptions leave consumers of family planning services at a disadvantage for accessing regular contraceptive methods or emergency contraceptives, therefore leading to an increased risk of unplanned pregnancy [4]. Our review suggests that an increase in unplanned pregnancies was seen during times of natural disaster. It is imperative that as relief efforts and funding are distributed across medical specialties, family planning services should be prioritized and considered as essential services. Furthermore, the overall use of contraceptives, including oral contraceptives and new IUDs, all decreased post-natural disaster pregnancies, further illustrating the need for family planning services to be accessible even during public health emergencies.

While the relationship between the COVID-19 pandemic and unintended pregnancy is still being determined, initial data shows a decreased use of contraceptives, an increase in the frequency of sexual intercourse, and a decrease in the desire to become pregnant [14]. These variables play a vital role in unintended pregnancy and have the potential to increase unintended pregnancy in those exposed to pandemics. Adverse outcomes, such as increases in neonatal morbidity, demonstrate the downstream effects of disruptions in prenatal care and the stressors of childbirth during natural disasters [16].

Some other considerations are the social and policy components within the society which the natural disaster or pandemic is affecting. Factors such as employment, socioeconomic status, social and political determinants of health, and family planning policies affect the outcomes noted in this review, that is, previous public health emergencies and COVID-19 continuing to impact public health [22]. Financial worries and economic instabilities influence a decreased desire for pregnancy while also creating additional barriers to obtaining contraception and family planning services [22].

Social, economic, and racial disparities in health are very apparent during the COVID-19 pandemic [22]. Policy improvements in these sectors are necessary along with those for family planning and contraceptive services. Thus far, in the United States during the COVID-19 pandemic, policy experts have classified family planning services are essential, which has allowed for further care through telemedicine, increased insurance coverage, and increased funding for family planning programs [22].

Limitations

The major limitation of this study is the small number of papers that met the inclusion criteria. Due to this reason, papers that met inclusion criteria were utilized regardless of rating or quality. Only English papers or those with English translations were included in this study further limiting the scope of the study to have a greater focus on European- or American-based studies. Additionally, some of the secondary and adverse outcomes studied in this paper may be due to the effects farther than those described, such as physiologic and complex medical issues of the birthing person prior to earthquake and hurricane exposure. Furthermore, the various natural disasters are occurring at various points in time, which may mean subjects have different desires or relationships with pregnancy and family planning services. Family planning and contraception policies in various countries at these various points in time were not addressed. The predictive COVID-19 data discussed varied based on algorithms or methods utilized leading to potential discrepancies in findings.

Implications

Of the 1.9 billion women of reproductive age worldwide, 1.1 billion require family planning services [23]. Family planning and obstetric providers should be aware of the unique challenges and limitations that accompany natural disasters and pandemics and find ways to facilitate care to prevent unintended pregnancy and adverse effects on the fetus and birthing person.

Currently, the CDC recommends that providers combat the decreased access to family planning services due to the COVID-19 pandemic by utilizing telehealth to see patients in a socially distanced manner [24]. However, providers must also keep the limitations and issues that accompany telehealth in mind. Factors such as access to a stable internet connection and the availability of webcams can make the utilization of telehealth more difficult, especially in socioeconomically disadvantaged communities, potentially creating a greater digital divide [25]. This application of telemedicine for contraceptive and family planning purposes should be prioritized and considered in future pandemics and natural disasters globally, given the increases in unplanned pregnancy rates during these times.

The CDC also recommends that family planning facilities focus on outreach to ensure that patients are aware of their options for acquiring these services [16]. Although CDC recommendations are localized to the United States, similar approaches can be taken worldwide. Other recommendations include utilizing pharmacist-prescribed contraception, prescribing a one-year supply of combined OCPs, and allowing patients to have an “advanced” or an “emergency” supply of OCPs and to utilize self-administered subcutaneous depot medroxyprogesterone acetate (DMPA) [26].

## Conclusions

Our review sheds light on the increased risk of unintended pregnancy that accompanies natural disasters and pandemics. This is an important area of research for any future natural disaster or pandemic. While theoretical data shows an increase in the negative outcomes for those seeking family planning services during the COVID-19 pandemic, more research should be done to elucidate the concrete relationship between COVID-19 and unintended pregnancy.
